# Relative and Absolute Interrater Reliabilities of a Hand-Held Myotonometer to Quantify Mechanical Muscle Properties in Patients with Acute Stroke in an Inpatient Ward

**DOI:** 10.1155/2017/4294028

**Published:** 2017-10-15

**Authors:** Wai Leung Ambrose Lo, Jiang Li Zhao, Le Li, Yu Rong Mao, Dong Feng Huang

**Affiliations:** ^1^Department of Rehabilitation Medicine, Guangdong Engineering and Technology Research Center for Rehabilitation Medicine and Translation, The First Affiliated Hospital, Sun Yat-sen University, Guangzhou 510080, China; ^2^Department of Electronic and Electrical Engineering, University College London, Torrington Place, London WC1E 7JE, UK

## Abstract

**Introduction:**

The reliability of using MyotonPRO to quantify muscles mechanical properties in a ward setting for the acute stroke population remains unknown.

**Aims:**

To investigate the within-session relative and absolute interrater reliability of MyotonPRO.

**Methods:**

Mechanical properties of biceps brachii, brachioradialis, rectus femoris, and tibialis anterior were recorded at bedside. Participants were within 1 month of the first occurrence of stroke. Relative reliability was assessed by intraclass correlation coefficient (ICC). Absolute reliability was assessed by standard error of measurement (SEM), SEM%, smallest real difference (SRD), SRD%, and the Bland-Altman 95% limits of agreement.

**Results:**

ICCs of all studied muscles ranged between 0.63 and 0.97. The SEM of all muscles ranged within 0.30–0.88 Hz for tone, 0.07–0.19 for decrement, 6.42–20.20 N/m for stiffness, and 0.04–0.07 for creep. The SRD of all muscles ranged within 0.70–2.05 Hz for tone, 0.16–0.45 for decrement, 14.98–47.15 N/m for stiffness, and 0.09–0.17 for creep.

**Conclusions:**

MyotonPRO demonstrated acceptable relative and absolute reliability in a ward setting for patients with acute stroke. However, results must be interpreted with caution, due to the varying level of consistency between different muscles, as well as between different parameters within a muscle.

## 1. Introduction

The mechanical properties of muscles such as tone, elasticity, and stiffness are often affected in patients with stroke [1]. Muscle tone is considered to be fundamental in maintaining balance, posture stability, and energy efficient muscle contractions [2]. The biceps brachii and brachioradialis are essential muscles that are frequently used in activities of daily living such as eating, dressing, and opening a door [3]. The rectus femoris, part of the quadriceps, is essential in gait and balance [4]. The tibialis anterior is a predictor of functional mobility of people with hemiplegia [5]. Since abnormal mechanical properties of these muscles might contribute to functional limitations and reduced mobility, changes in mechanical properties are routinely monitored as part of rehabilitation programs [6]. Muscle mechanical properties such as tone and stiffness are clinically assessed subjectively by scoring resistance to passive motion on scales such as the modified Ashworth scale (MAS) or by manual palpation [7]. However, the appropriateness of using the MAS to measure muscle spasticity has been criticized in the literature [8]. Laboratory techniques such as ultrasound imaging with dynamometry [9] and magnetic resonance elastography [10] and investigations using a joint torque servo motor [11] are not clinically feasible. Therefore, objectively quantifying changes in muscle tone and other muscle mechanical properties continues to be a challenge. A commercially available hand-held device known as MyotonPRO was first made available a decade ago as a mean of objectively measuring mechanical muscle properties. The technology works on the principle of applying multiple short impulses over the muscle bulk via the testing probe [12]. The impulses cause oscillations in the muscle tissue. The acceleration transducer records the oscillation waveform, which is then used to calculate the parameters of tone, decrement, stiffness, and creep. The validity of the technology used in a myotonometer has been investigated in several studies [11, 13, 14]. A recently published study validated the myotonometric measurement of the elbow flexor against a stretch technique that measures changes in resistance torque during repeated joint rotations controlled by a servomotor [11]. The results showed that myotonometry and conventional passive stretch techniques could identify substantial changes in spastic muscles. Fröhlich-Zwahlen et al. (2014) [13] investigated the validity of the lower limb muscles of chronic stroke patients with limited hypertonia by comparing the parameters with muscle thickness and muscle strength measured by ultrasound. The results revealed that muscle strength and thickness were positively correlated with stiffness and tone. The tone and stiffness of hand muscles were also shown to be significantly correlated with hand strength and upper limb motor function [14]. Other published studies have also reported statistically significant differences in mechanical muscle properties between different age groups of women [15] and in mixed populations [16] between people with Parkinson's disease and healthy individuals [17] and between people with chronic stroke and healthy individuals [18] when measured using a myotonometer. Existing evidence suggests that myotonometry is a valid technology to record the mechanical properties of muscles.

The latest myotonometer model known as MyotonPRO has an embedded triaxial accelerometer that enables measurements to be taken in different postures and positions [19]. This feature should in theory improve its reliability, particularly in a ward setting where there may be less room to maneuver the patients into the required position and where patients often have reduced mobility (e.g., patients with acute stroke). Despite the theoretical advantage of the MyotonPRO, there is limited evidence to demonstrate its reliability when used in a ward setting. The majority of reliability studies of the MyotonPRO focused on the healthy population [16, 19, 20]. Studies that investigated the reliability of previous models (Myoton 3) in patients with subacute and chronic stroke [21, 22] indicated good inter- and intrarater reliability. However, both studies were conducted in laboratory settings rather than in a clinical setting. Practitioners who operate hand-held measuring devices in a clinical setting face additional challenges such as time pressure or the inability to place patients in an ideal position. Other authors have also reported environmental factors such as background noise that was present in a clinical environment that may possibly influence muscle tone in patients with pathology [7]. Previous studies have indicated that the reliability of hand-held measuring device might be affected by the operator's experience [23] and measuring technique [24] and in pathological groups [25]. Therefore, it cannot be assumed that using the device in a ward setting would yield the same reliability as in a laboratory setting. The reliability of MyotonPRO when used in a clinical setting must be established if it was to be used as outcome measure to monitor the effects of interventions.

The aims of this study were to assess the relative and absolute interrater reliabilities of MyotonPRO when it was used in a ward setting in an acute stroke population. This study was among the first to assess the reliability of the device in patients with acute stroke.

## 2. Material and Methods

### 2.1. Study Setting

This single-center study was conducted in a university-affiliated hospital. Participants were recruited from the inpatient rehabilitation ward. Measurements were taken in the ward at the participant's bedside by two physiotherapists. The assessors underwent 4 hours of training from the manufacturer and an additional 2 days of practice with the MyotonPRO.

### 2.2. Recruitment

This study was part of a larger trial that investigated the effect of multisensory interactive training in patients with acute stroke. Patients who were admitted to the inpatient ward were screened for eligibility as part of a routine assessment. Baseline information such as age, gender, height, weight, affected side, muscle tone (assessed by modified Ashworth scale), type of stroke, range of movement at all joints, ability to follow instructions, bedside mobility, and walking ability (10-meter walk test) were collected as part of routine clinical assessment. Suitable participants were identified by the clinical team and given an information sheet about the study. These potential participants were then approached by a member of the research team to inquire if they were interested and willing to take part in the study. A screening log of all of the nonrecruited patients and the reason for exclusion was maintained.

### 2.3. Sample Population

The inclusion criteria were as follows: (1) the participant's first stroke that occurred less than a month ago; (2) stage 2 or above of Brunnstrom classification at upper extremity, hand, or lower extremity; (3) MRI or CT confirmed stroke; (4) age between 40 and 80; (5) the patient having at least 20 degrees of wrist flexion/extension and at least 10 degrees of finger flexion and extension at the affected limbs; (6) ability to walk at least 10 meters with or without assistance; (7) no severe cognitive impairment (Mini-Mental State Examination score less than 10 [26]).

This study excluded participants who were medically unstable or suffered from brain stem injury.

### 2.4. Ethics

The study was approved by the Medical Ethical Committee of the First Affiliated Hospital of Sun Yat-sen University in Guangzhou, China (ethics number [2014]88). All of the patients who satisfied the criteria were invited to take part in the study. Patients were given an information sheet regarding the study and had time to consider whether they wished to take part in the trial, and the patients were encouraged to ask questions prior to participating. Written informed consent was obtained from all of the patients who agreed to take part, and the participants could withdraw from the trial at any time without giving a reason.

### 2.5. Instrument

A MyotonPRO was used to measure the muscle properties of each participant's affected and nonaffected side. Although the triaxial accelerometer allows the device to be held in any direction when taking a measurement, muscle properties may be affected by gravity. The testing end of the MyotonPRO was placed perpendicular to the middle of the muscle belly being tested. The probe was pushed against the skin to the required depth (marked as a red line on the probe; the indicator changed from red to green). The device was set in triple scan mode, which consisted of three consecutive impulses, one second apart. There was no consensus for the optimum number of impulses needed for reliable measurement without compromising time-effectiveness. Triple scan mode was selected in this study to enable comparison with the two published studies on the stroke population that also used tripe scan mode [21, 22]. Values for mean, standard deviation, and the coefficient of variation for the three measurements were displayed on the screen. Any measurement set that had a coefficient of variation of over 3% was erased and remeasured. This methodology is recommended standard operating procedure and is commonly used in the published literature [7, 19, 27].

### 2.6. Parameters

**Figure 1:**
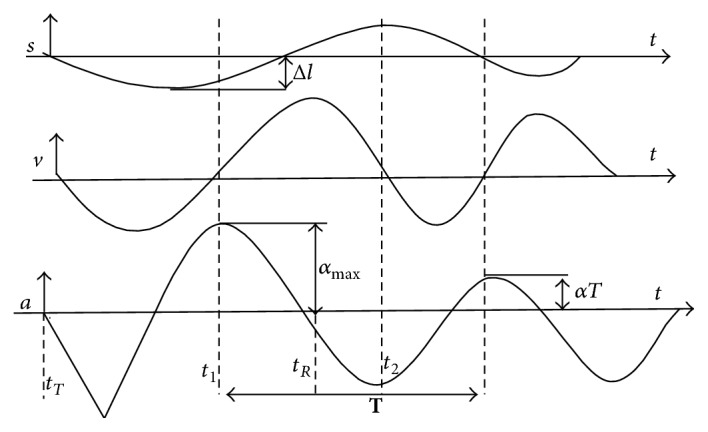
Oscillation graph that illustrates the calculation for each parameter.* s*: displacement;* v*: velocity;* a*: acceleration;  Δ*l*: the deformation depth of muscle mass; *α*_max_: maximal amplitude of oscillation.

Parameters that were recorded were muscle tone, decrement, stiffness, and creep. The natural oscillation frequency (Hz) that characterizes muscle tone is calculated as Hz = 1/*T*, where *T* is the oscillation period in seconds (Figure 1). The logarithmic decrement of the damping frequency that characterizes muscle elasticity is calculated as decrement = ln⁡(*α*_max_/*α*), where *α*_max_ is the maximal amplitude of oscillation and *αT* is the amplitude of the second oscillation cycle. The maximal amplitude of oscillation that characterizes muscle stiffness (N/m) is calculated as stiffness =  *α*_max_*∗m*_probe_/Δ*l*, where Δ*l* is the deformation depth of muscle mass and *m*_probe_ is the mass of the testing probe. Creep is the ratio of relaxation time to deformation, which is calculated as creep = *R*/(*t*_1_ − *t*_*T*_), where *R* is the mechanical stress relaxation time (ms): *R* = *t*_*R*_ − *t*_1_.

**Figure 2:**
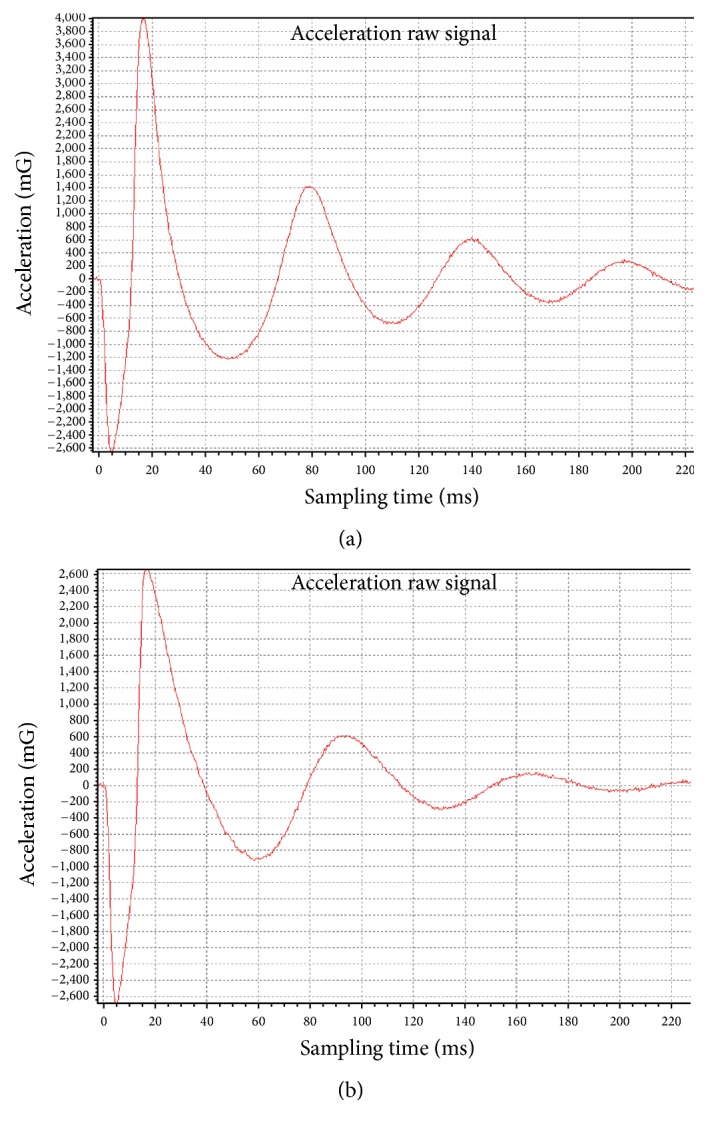
Illustrations of the raw acceleration traces. (a) Acceleration trace of rectus femoris. Frequency = 16.98 Hz; decrement = 1.02; stiffness = 370 M/m; creep: 0.85 (*R* = 14 ms). (b) Acceleration trace of biceps brachii. Frequency = 14.45 Hz; decrement = 1.39; stiffness = 238 N/m; creep = 1.34 (*R* = 22.3 ms).

Figure 2 shows an example of the raw acceleration trace recorded at the rectus femoris and biceps brachii. It illustrates differences in the acceleration curve as the values of the measured variables change. It can be seen that the natural oscillation frequency of the rectus femoris within the first second is higher than that of the biceps brachii, therefore yielding a higher value for muscle tone for the rectus femoris. The amplitudes of *αT* oscillation of the first and second cycles are higher in the rectus femoris than in the biceps brachii, yielding a lower value of decrement. Since *α*_max_ is higher in the rectus femoris, this muscle is associated with a higher stiffness value.

### 2.7. Procedure

The testing protocol was designed to be replicated in routine clinical practice. Therefore, participants were advised to continue with their normal routine without controlling their physical activity level. This study focused on the biceps brachii, brachioradialis, rectus femoris, and tibialis anterior muscles. All of the tested muscles have been validated in previous studies [13, 28–30]. Reliability was performed as part of the baseline assessment for the main study. The location of the testing sites was in accordance with the standard operating procedure suggested by the manufacturer [12] for MyotonPRO. For the biceps brachii measurements, participants were positioned in a supine position with their elbow supinated and flexed to 10–15 degrees, allowing relaxation of the muscle [31]. A towel was placed at the wrist to support the arm in a flexed position. The testing site of the biceps brachii was located using a measuring tape to identify the halfway point between the anterior aspect of the lateral tip of the acromion and the medial boarder of the cubital fossa; this point was marked with a pen. When measuring the brachioradialis, the elbow was in an extended position with the forearm pronated. The test site was located by identifying the upper two-thirds distance from the lateral supracondylar ridge to the styloid process. The rectus femoris measurement was performed in a supine position with the hips in a neutral position and the knees fully extended. Measurements were taken at two-thirds of the distance between the anterior superior iliac spine and the superior pole of the patellar. Measurements of the tibialis anterior were taken at the upper two-thirds of the distance between the lateral condyle of the tibia and the medial cuneiform. Measurements were taken of the biceps brachii, brachioradialis, rectus femoris, and tibialis anterior, in that order, beginning from the left side. The entire session was repeated 15 minutes later by the second rater who followed the same procedure. The order of the raters was not randomized with rater 1 always performing the first measurement followed by rater 2. Participants were asked to remain in a lying position and relaxed for 15 minutes after the first measurement.

### 2.8. Data Analysis

Statistical analyses were performed using SPSS Statistics 20 software (IBM, United States). Data normality was assessed using the Kolmogorov-Smirnov test and frequency histograms. Intraclass correlation coefficient (ICC) was used to determine the relative reliability. The demographics of the sample population were assessed using descriptive statistics. The ICC model of (3, *k*) was used to assess the relative interrater reliability. The interpretation of ICC was based on the recommendation by Domholdt [32]: 1.00–0.9 = very high; 0.89–0.7 = high; 0.69–0.50 = moderate; 0.49–0.26 = low; <0.26 = poor. Absolute reliability was assessed by standard error of measurements (SEM), SEM%, the smallest real difference (SRD), and SRD% [33]. SEM% of less than 10% may be considered small [34] and SRD% of below 30% may be considered acceptable [35]. Bland and Altman analysis was used to identify systematic bias and 95% limits of agreement [36] on the pooled data for each muscle group.

## 3. Results

Twenty-nine participants with acute stroke were recruited for this study from a single center. Participants cooperated well during the data collection session. The mean age of the sample population was 58 years. A summary of the demographics of the sample population is listed in Table 1. Descriptive statistics of the myotonometric measurements of the two sessions are listed in Table 2.

### 3.1. Relative Reliability

The results of relative reliability assessed by ICCs are presented in Table 2. On the affected side, the ICCs for biceps brachii, brachioradialis, and tibialis anterior range within 0.76–0.89, 0.90–0.93, and 0.83–0.91, respectively. This indicates high to very high consistency and agreement between the two measurements of those muscles. Rectus femoris on the affected side has high consistency and agreement between the two measurements for tone, stiffness, and creep whereas moderate consistency was observed for decrement. On the nonaffected side, biceps brachii and brachioradialis have moderate to high consistency as ICCs range within 0.66–0.74 and 0.65–0.88, respectively. Rectus femoris has very high consistency between the two measurements in all four parameters (0.96–0.99). Tibialis anterior has ICC range within 0.65–0.91 which indicates moderate to very high consistency.

### 3.2. Absolute Reliability

The mean differences between each assessor for the tested muscle groups are shown in Table 2. A summary of the absolute reliability is presented in Table 3. The SEM of all of the muscle groups ranged within 0.30–0.88 Hz for tone, 0.07–0.19 for decrement, 6.42–20.20 N/m for stiffness, and 0.04–0.07 for creep. The SRD of all of the muscle groups ranged within 0.70–2.05 Hz for tone, 0.16–0.45 for decrement, 14.98–47.15 N/m for stiffness, and 0.09–0.17 for creep. The 95% limits of agreement indicated small error bands for all parameters within each muscle group, except for stiffness.  Table 4 indicates the results of 95% limits of agreement for the affected and nonaffected side.

**Figure 3:**
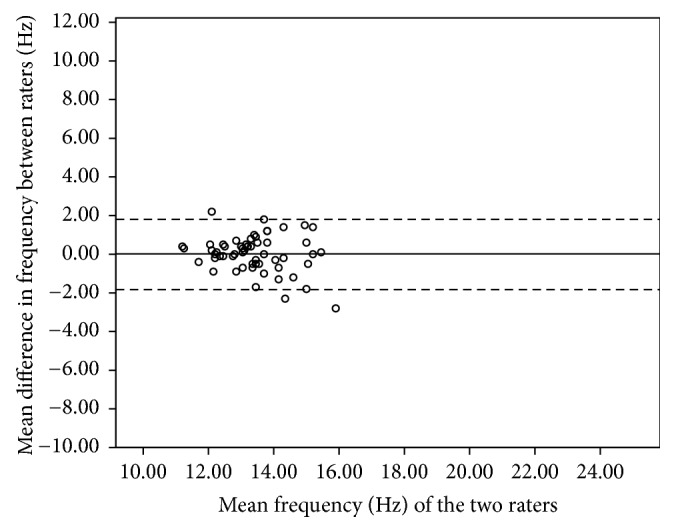
Bland and Altman plot of pooled frequency of biceps brachii.

**Figure 4:**
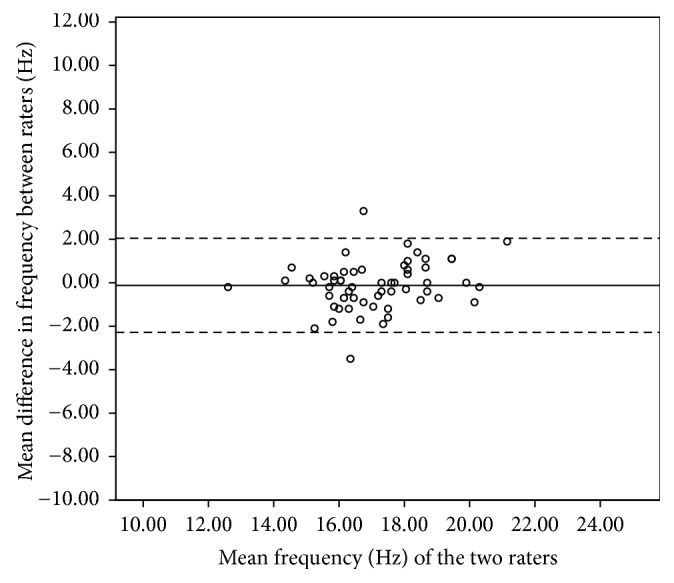
Bland and Altman plot of pooled frequency of brachioradialis.

**Figure 5:**
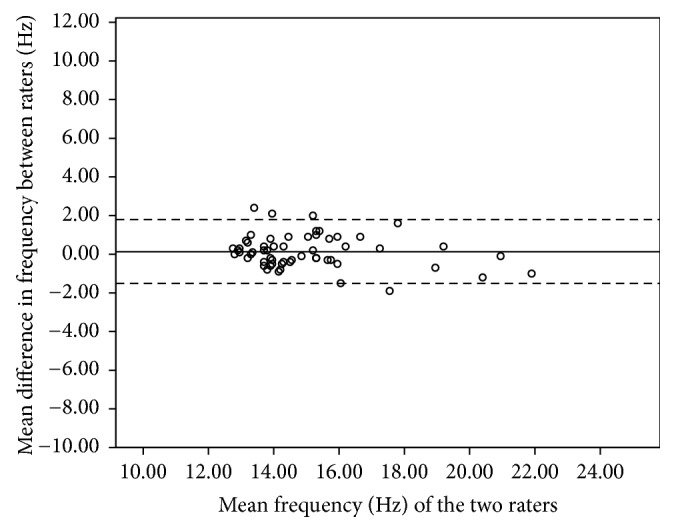
Bland and Altman plot of pooled frequency of rectus femoris.

**Figure 6:**
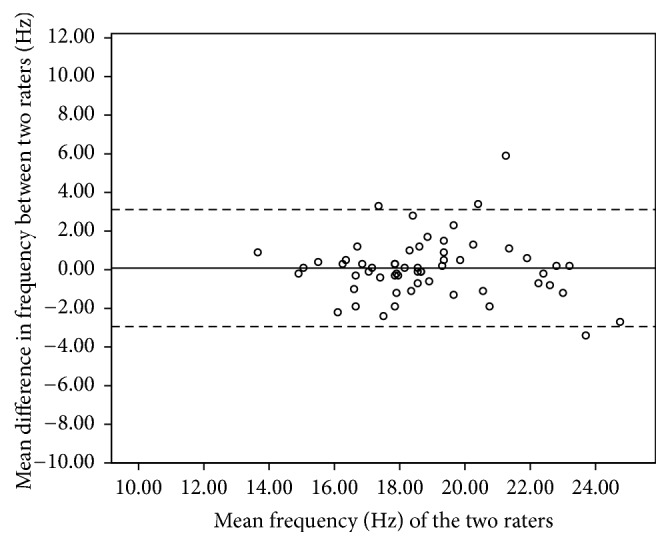
Bland and Altman plot of pooled frequency of tibialis anterior.

The 95% limits of agreement for the pooled data of biceps brachii ranged from +1.8 to −1.8 Hz for frequency, +0.32 to −0.26 for decrement, +41.16 to −35.79 N/m for stiffness, and 0.24 to −0.27 for creep. The 95% limits of agreement for the pooled data of the brachioradialis ranged from +2.05 to −2.28 Hz for frequency, +0.37 to −0.39 for decrement, +50.29 to −70.11 N/m for stiffness, and 0.24 to −0.17 for creep. For the rectus femoris, the 95% limits of agreement ranged from +1.78 to −1.54 Hz for frequency, +0.41 to −0.32 for decrement, +38.86 to −35.93 N/m for stiffness, and 0.16 to −0.19 for creep. For the tibialis anterior, the 95% limits of agreement ranged from +3.11 to −2.95 Hz for frequency, +0.69 to −0.48 for decrement, +75.54 to −58.34 N/m for stiffness, and 0.168 to −0.22 for creep. Figures 3–6 illustrate the Bland and Altman plots for muscle tone of the pooled data of all tested muscles.

No systematic bias was identified from the Bland and Altman plots for all tested muscles in all parameters. Figures 3–6 illustrate the Bland and Altman plots for muscle tone of the pooled data for all tested muscles.

## 4. Discussion

The present study was among the first to examine the interrater reliability of MyotonPRO technology when applied in a ward setting in patients with acute stroke. Findings of the present study provide comparative reference data from patients with acute stroke and form the basis for MyotonPRO technology to be used as a clinical outcome measure tool.

### 4.1. Relative Reliability

The ICC analysis indicated moderate to very high relative reliability for measuring the biceps brachii, brachioradialis, rectus femoris, and tibialis anterior in patients with acute stroke in a ward setting. Each parameter within a particular muscle and between different muscle groups demonstrated varying levels of consistency. The variations in reliability of different parameters within a muscle observed in this study were consistent with a published study of stroke patients with an early model of the Myoton-3 [22]. They reported that ICC ranged between 0.72 and 0.94 in patients with subacute stroke for the muscle biceps brachii. A recent study published by Van Deun et al. [7] assessed interrater reliability in older adults with paratonia. They reported that the reliability of each parameter ranged from moderate to high (ICC: 0.43 for tone, 0.62 for decrement, and 0.73 for stiffness) for the biceps brachii muscle in the pathological group. Similar findings were reported in the literature for a previous model of Myoton devices. Bizzini and Mannion [37] reported the ICC for the rectus femoris of 0.85 but only 0.4 for the vastus lateralis with the Myoton-2. A plausible explanation for the variability in interrater reliability of different muscles is the variable distribution of subcutaneous fat, which affects wave attenuation.

When comparing between affected and nonaffected side, it could be seen that the biceps brachii and brachioradialis were less reliable on the nonaffected side. Chuang et al. [22] also reported a lower ICC for biceps brachii tone on the nonaffected side of subacute stroke patients which was consistent with the findings of this study.

The relative reliability index observed in this study suggested that the MyotonPRO may be a reliable instrument for use in a ward setting. However, measurements recorded from the device should be interpreted with caution as results from this study suggested that the reliability might not be the same for all muscle groups and for all parameters.

### 4.2. Absolute Reliability

The ICC is easily influenced by between-subject variance and must be complemented by the absolute reliability index [38]. Data for the biceps brachii were available from two studies on the chronic stroke population for direct comparison. The SEM and SEM% values of bilateral biceps brachii tone and stiffness were higher than those reported by Chuang et al. (2013) [21] in the subacute stroke population but were similar to those reported by Chuang et al. (2012) [22] in the chronic stroke population. The SRD values for the affected side biceps brachii tone and stiffness observed in this study were higher than those reported in subacute stroke population [21] but were lower than those reported in chronic stroke population [22]. The SEM% and SRD% were comparable between the affected and the nonaffected side in all parameters of the studied muscles, except for rectus femoris. The largest between-side differences were observed in decrement and stiffness of rectus femoris where SEM% and SRD% of the affected side were approximately three times higher than the nonaffected side. The SEM% was over 10% for decrement of bilateral tibialis anterior and on the affected side of rectus femoris. This finding suggested that the parameter of decrement may not be sensitive to detecting small changes. SRD% of decrement of tibialis anterior on the nonaffected side of was over 30% which also suggests low reproducibility. Although SEM% and SRD% are useful indices, however, the interpretation of SEM% and SRD% must be cautious due to lack of standardized interpretation. The suggested cut-off value for SEM% of less than 10% and SRD% of below 30% is rather arbitrary and thus has limited generalizability beyond the specific studies.

The SEM and SRD of tone, decrement, and stiffness for rectus femoris on the affected and nonaffected side were higher than those reported in healthy older males [20]. These findings suggest that measurements recorded by MyotonPRO contain more error variability around the mean and are less sensitive to change when used in patients with acute stroke in a ward than in healthy individuals in laboratory setting to measure mechanical properties of rectus femoris. Therefore, a larger change was required to be deemed “real” if the device was to be used in a ward setting. The results of this study can be used as a reference for the measurement error of the MyotonPRO to determine the real change between repeated measurements for patients with acute stroke.

### 4.3. Bland and Altman Analysis

To date, only a few studies have calculated the 95% limits of agreement. The purpose of the 95% limits of agreement is to provide a range of error that may relate to clinical acceptability [38]. The error range of limits of agreement varies across different muscles, which is consistent with the findings from our relative reliability analysis. Therefore, clinicians should be cautious when interpreting results and must be aware that different muscle group may have different error ranges.

Bland and Altman analysis indicated no systematic bias since all of the measurements included zero. For the biceps brachii muscle, the ranges of the 95% limits of agreement on the affected side for tone, decrement, and stiffness were consistent with those reported by Chuang et al. (2013) in the chronic stroke population with Myoton-3. This finding suggests that the range of error was not affected when the device was used in a ward setting for measuring the biceps brachii. When compared with the limits of agreement of the rectus femoris recorded in healthy participants with MyotonPRO [20], the limits of agreement of the three parameters (tone, decrement, and stiffness) spanned a larger range of values in our study. The wider error of limits of agreements observed in our study is consistent with the findings on SRD of the rectus femoris in which a larger change in measurement would be required to be deemed a real change. Therefore, using the device to measure the rectus femoris in a ward setting may not be as reliable as in the laboratory setting. Although the 95% limits of agreement give an indication of the range of error, there is currently insufficient published data about the four parameters of tone, decrement, stiffness, and creep to determine whether the observed error range in this study is clinically acceptable. The results from this study provide reference data for the MyotonPRO that can be used to monitor the effects of interventions.

### 4.4. Limitations

One of the limitations of this study was that variables that might influence muscle tone, stiffness, and elasticity, such as age, ambient and body temperature, subcutaneous soft tissue, and the degree of physical exercise that patients received on the day of data collection, were not controlled. However, this fact should not affect the reliability analysis since the readings were compared between raters rather than between participants. Another limitation was that the state of the muscles at the time of data collection was not objectively recorded. Therefore, it was not possible to be certain that a participant's muscles were in a resting state, nor were the states of the muscle exactly the same between the 2 measurements. Agyapong-Badu et al. (2013) suggested a 10-minute relaxation period prior to recording. Thus this study included 15-minute gap between the two measurements to enable the muscle to relax and return to its previous state. The 15-minute rest between the two measurements may also be a source of variability since there was no relaxation period prior to the first set of measurements. This study was not specifically set out to test the reliability of the device on a range of participants with different spasticity levels. This may limit the generalizability of the current findings. However, the primary aim of this study was to establish the interrater variability of the device when used in a ward setting in people with acute stroke. Additional studies are required to assess the reliability of the myotonometer across a range of stroke patients with different levels of spasticity in a ward setting. The reliability analysis in this study was not corrected for BMI. Although there is concern that subcutaneous fat may affect the myotonometer readings, however, two studies previously reported low to moderate correlation between the amount of subcutaneous fat and muscle parameters [13, 16]. This study followed the standard operating procedure recommended by the manufacturer to rerecord the measurement sets that contained values that exceeded 3% of the coefficient of variation. This procedure makes the results appear more favorable. However, this practice is common among published studies that have used a myotonometer. Additional investigations are required to establish the number of trials that were disregarded due to a high coefficient of variation. This study used a pen to mark the spot to guide the raters in obtaining measurements. The rationale behind using a pen to mark the test site was to minimize confounding factors related to the repeatability of test site identification. This study specifically assessed the reliability of the device when used in a ward setting. It is feasible to leave a small mark on the surface of the skin during the inpatient stay to enable multiple test procedures. Additional investigation may be beneficial to assess the reliability of the device when the test site is not marked.

## 5. Conclusions

The study has demonstrated that the MyotonPRO has acceptable relative and absolute interrater reliabilities when measuring mechanical muscle properties in a ward setting. Agreement between raters measurement was high with low measurement errors. Although the MyotonPRO is a more useful instrument for objectively quantifying muscle properties than subjective scales, one must be cautious when interpreting the results since reliability of the device does not appear to be consistent throughout all muscle groups and within all the parameters it measures. Further research to understand the validity of myotonometric measures in a ward setting is recommended.

## Figures and Tables

**Table 1:** A summary of the demographics of all participants. stdv: standard deviation.

Characteristic	Range	Mean (stdv)
Age	30–83	58.9 (12.6)
Dominant side, left/right	—	29/0
Affected side, left/right	—	14/15
Gender, male/female	—	24/5
Body Mass Index	10.82–30.12	23 (4)
Days after stroke onset	9–30	20 (7.16)

Brunnstrom classification	Range	(Median, mode)

Upper extremity	1–5	3 (2)
Hand	1–5	2 (1)
Lower extremity	1–5	3 (3)

Modified Ashworth scale	Range	(Median, mode)

Biceps brachii	0–2	1 (2)
Brachioradialis	0–2	1 (2)
Rectus femoris	0–2	1 (2)
Tibialis anterior	0-1	0 (1)

**Table 2:** The mean between rater differences and ICC indexes. Mean *d*: mean difference between first and second measurements; CI: confidence interval.

Interrater reliability					
	Parameters	Rater 1	Rater 2	Mean *d*	ICC (95% CI)
Biceps brachii (affected)	Frequency (Hz)	13.31 (1.13)	13.11 (1.43)	0.20	0.83 (0.63–0.92)
	Decrement	1.27 (0.2)	1.24 (1.24)	0.04	0.89 (0.77–0.95)
	Stiffness (N/m)	220.55 (18.66)	215.52 (27.03)	5.03	0.76 (0.48–0.89)
	Creep	1.45 (0.16)	1.48 (0.18)	−0.03	0.81 (0.60–0.91)

Brachioradialis (affected)	Frequency (Hz)	13.91 (1.70)	16.94 (1.79)	−3.03	0.93 (0.86–0.97)
	Decrement	1.20 (0.19)	1.44 (0.31)	−0.24	0.91 (0.80–0.96)
	Stiffness (N/m)	231.66 (54.26)	330.86 (57.05)	−99.21	0.92 (0.83–0.96)
	Creep	1.39 (0.21)	1.02 (0.16)	0.37	0.90 (0.80–0.96)

Rectus femoris (affected)	Frequency (Hz)	14.73 (1.67)	15.08 (2.46)	−0.35	0.86 (0.71–0.93)
	Decrement	1.61 (0.45)	1.67 (0.29)	−0.06	0.65 (0.26–0.84)
	Stiffness (N/m)	287.86 (41.43)	301.24 (67.07)	−13.38	0.88 (0.74–0.95)
	Creep	1.29 (0.18)	1.26 (0.25)	0.03	0.87 (0.73–0.94)

Tibialis anterior (affected)	Frequency (Hz)	18.81 (2.16)	18.37 (2.43)	0.44	0.91 (0.82–0.96)
	Decrement	1.26 (0.42)	1.11 (0.35)	0.15	0.83 (0.64–0.92)
	Stiffness (N/m)	376.00 (53.23)	354.83 (58.06)	21.17	0.91 (0.82–0.96)
	Creep	0.98 (0.18)	1.05 (0.19)	−0.07	0.89 (0.77–0.95)

Biceps brachii (nonaffected)	Frequency (Hz)	13.50 (0.98)	13.67 (0.94)	−0.17	0.70 (0.36–0.86)
	Decrement	1.21 (0.2)	1.19 (0.14)	0.02	0.74 (0.46–0.88)
	Stiffness (N/m)	218.83 (18.99)	223.72 (18.90)	−4.9	0.66 (0.29–0.84)
	Creep	1.44 (0.14)	1.44 (0.14)	0.00	0.70 (0.35–0.86)

Brachioradialis (nonaffected)	Frequency (Hz)	17.16 (1.99)	17.41 (1.32)	−0.25	0.78 (0.54–0.90)
	Decrement	1.36 (0.23)	1.30 (0.21)	0.06	0.65 (0.25–0.84)
	Stiffness (N/m)	323.00 (57.98)	323.76 (47.64)	−0.76	0.88 (0.74–0.97)
	Creep	1.04 (0.15)	1.02 (0.11)	0.02	0.71 (0.39–0.87)

Rectus femoris (nonaffected)	Frequency (Hz)	14.84 (1.64)	14.93 (1.88)	−0.09	0.97 (0.94–0.99)
	Decrement	1.59 (0.46)	1.69 (0.34)	−0.1	0.96 0.92–0.98)
	Stiffness (N/m)	288.83 (41.15)	292.03 (48.60)	−3.21	0.99 (0.97–0.99)
	Creep	1.28 (0.17)	1.28 (0.21)	0	0.96 (0.92–0.98)

Tibialis anterior (nonaffected)	Frequency (Hz)	18.96 (2.43)	19.23 (2.61)	−0.27	0.87 (0.72–0.94)
	Decrement	1.21 (0.34)	1.15 (0.28)	0.06	0.65 (0.27–0.83)
	Stiffness (N/m)	368.4 (51.97)	372.41 (50.03)	−3.97	0.83 (0.63–0.92)
	Creep	1.00 (0.16)	0.98 (0.17)	0.02	0.91 (0.81–0.96)

**Table 3:** A summary of absolute reliability indexes. SEM: standard error measurement; SRD: smallest real difference.

Muscles	Parameters	Affected		Nonaffected		Affected		Nonaffected	
		SEM	SEM%	SEM	SEM%	SRD	SRD%	SRD	SRD%
Biceps brachii	Frequency (Hz)	0.49	3.73%	0.45	3.31%	1.14	8.71%	1.06	7.71%
	Decrement	0.08	6.22%	0.07	6.19%	0.18	14.50%	0.17	14.45%
	Stiffness (N/m)	10.11	4.70%	9.35	4.21%	23.60	10.97%	21.81	9.82%
	Creep	0.07	4.57%	0.06	4.39%	0.16	10.66%	0.15	10.23%

Brachioradialis	Frequency (Hz)	0.48	2.85%	0.61	3.55%	1.12	6.65%	1.43	8.28%
	Decrement	0.11	7.59%	0.10	7.61%	0.26	17.72%	0.23	17.76%
	Stiffness (N/m)	20.20	6.18%	15.78	4.96%	47.15	14.43%	36.83	11.58%
	Creep	0.05	4.56%	0.06	5.71%	0.11	10.63%	0.14	13.32%

Rectus femoris	Frequency (Hz)	0.73	4.92%	0.30	2.00%	1.71	11.47%	0.70	4.68%
	Decrement	0.19	11.77%	0.07	4.02%	0.45	27.46%	0.16	9.38%
	Stiffness (N/m)	18.19	6.18%	6.42	2.16%	42.46	14.41%	14.98	5.05%
	Creep	0.07	5.67%	0.04	3.05%	0.17	13.23%	0.09	7.13%

Tibialis anterior	Frequency (Hz)	0.63	3.46%	0.88	4.52%	1.47	8.08%	2.05	10.56%
	Decrement	0.14	12.22%	0.17	14.03%	0.33	28.51%	0.39	32.73%
	Stiffness (N/m)	14.76	4.15%	19.60	5.15%	34.44	9.69%	45.73	12.02%
	Creep	0.05	5.16%	0.05	5.09%	0.13	12.04%	0.11	11.87%

**Table 4:** A summary of limits of agreement between the two measurements.

Muscle group	Parameters	Affected			Nonaffected		
		Upper LOA	Lower LOA	Range	Upper LOA	Lower LOA	Range
Biceps brachii	Frequency (Hz)	1.9	−1.98	3.88	1.88	−1.7	3.58
	Decrement	0.33	−0.28	0.61	0.33	−0.24	0.57
	Stiffness (N/m)	39.5	−40.81	80.31	41.00	−31.55	72.55
	Creep	0.25	−0.28	0.53	0.24	−0.26	0.50

Brachioradialis	Frequency (Hz)	1.87	−1.97	3.84	2.23	−2.61	4.84
	Decrement	0.38	−0.39	0.77	0.38	−0.4	0.78
	Stiffness (N/m)	54.17	−70.59	124.76	47.75	−70.51	118.26
	Creep	0.22	−0.16	0.38	0.27	−0.18	0.45

Rectus femoris	Frequency (Hz)	2.49	−3.19	5.68	1.31	−1.02	2.33
	Decrement	0.7	−0.82	1.52	0.26	−0.28	0.54
	Stiffness (N/m)	54.6	−81.36	135.96	22.54	−22.06	44.6
	Creep	0.31	−0.25	0.56	0.38	−0.39	0.77

Tibialis anterior	Frequency (Hz)	2.18	−2.74	4.92	3.85	−2.96	6.81
	Decrement	0.63	−0.45	1.08	0.76	−0.52	1.28
	Stiffness (N/m)	59.7	−57.83	117.53	88.40	−55.84	144.24
	Creep	0.2	−0.22	0.42	0.15	−0.22	0.37
